# Is ^131^I ablation necessary for patients with low-risk papillary thyroid carcinoma and slightly elevated stimulated thyroglobulin after thyroidectomy?

**DOI:** 10.1590/2359-3997000000158

**Published:** 2016-01-01

**Authors:** Pedro Weslley Rosario, Gabriela Franco Mourão

**Affiliations:** 1 Santa Casa de Belo Horizonte Belo Horizonte MG Brazil Santa Casa de Belo Horizonte, Belo Horizonte, MG, Brazil

**Keywords:** Thyroid cancer, low-risk, postoperative thyroglobulin, radioiodine, recurrence

## Abstract

**Objective:**

This prospective study evaluated the recurrence rate in low-risk patients with papillary thyroid cancer (PTC) who presented slightly elevated thyroglobulin (Tg) after thyroidectomy and who did not undergo ablation with ^131^I.

**Subjects and methods:**

The study included 53 low-risk patients (nonaggressive histology; pT1b-3, cN0pNx, M0) with slightly elevated Tg after thyroidectomy (> 1 ng/mL, but ≤ 5 ng/mL after levothyroxine withdrawal or ≤ 2 ng/mL after recombinant human TSH).

**Results:**

The time of follow-up ranged from 36 to 96 months. Lymph node metastases were detected in only one patient (1.9%). Fifty-two patients continued to present negative neck ultrasound. None of these patients without apparent disease presented an increase in Tg.

**Conclusions:**

Low-risk patients with PTC who present slightly elevated Tg after thyroidectomy do not require ablation with ^131^I.

## INTRODUCTION

The need for radioiodine is controversial in patients with papillary thyroid cancer (PTC) who underwent apparently complete tumor resection and who show no signs of persistent disease after surgery. Patients with intrathyroid microcarcinoma and nonaggressive histology do not benefit from ablation with ^131^I (1-3). In contrast, adjuvant radioiodine therapy is recommended for patients who are at high risk of recurrence (2,3). The controversy surrounds patients who are at low risk of recurrence. In these patients, stimulated postoperative thyroglobulin (Tg) ≤ 1 ng/mL has been proposed as a criterion to select subjects who do not require ablation (4-9). It is known that persistent disease is rare if stimulated Tg is ≤ 1 ng/mL in the absence of anti-Tg antibodies (TgAb) (5,6) and, even without ablation, recurrence rarely occurs (4,7-9).

It is possible that tumor persistence and recurrence are also uncommon in low-risk patients with only slightly elevated stimulated Tg after surgery. The few cases of metastases observed at these Tg concentrations involve the cervical lymph nodes and can be detected by ultrasonography (US) (5,10,11). In fact, low-risk patients with only slightly elevated stimulated Tg and US showing no abnormalities rarely exhibit ectopic uptake upon post-therapy whole-body scanning (RxWBS) (5,11). Furthermore, two studies reported tumor recurrence in only 1/61 patients with Tg between 1 ng/mL and 5 ng/mL after levothyroxine (L-T4) withdrawal who did not receive ^131^I (4,9).

The objective of this prospective study was to evaluate the recurrence rate in low-risk patients who presented slightly elevated stimulated Tg [> 1 ng/mL, but ≤ 5 ng/mL after L-T4 withdrawal or ≤ 2 ng/mL after recombinant human TSH (rhTSH)] and negative neck US after thyroidectomy and who did not undergo ablation with ^131^I.

## MATERIALS AND METHODS

### Design

Prospective study.

### Patients

Patients consecutively seen at our institution who met the following criteria were first selected: diagnosis of PTC; submitted to total thyroidectomy with apparently complete tumor resection; no signs of persistent disease after surgery, and classified as low risk of recurrence. Patients presenting one of the following characteristics were excluded because of a higher risk of recurrence: tumor > 4 cm and extrathyroid invasion (both); extensive extrathyroid invasion (pT4), irrespective of tumor size; aggressive histological subtype (*e.g*., tall-cell, columnar-cell, diffuse follicular variant) or vascular invasion; lymph node metastases detected by preoperative US or suspected during surgery (cN1). The patients were not submitted to elective central compartment lymph node dissection. Patients with intrathyroid microcarcinoma, and patients with the noninvasive encapsulated follicular variant of papillary carcinoma, who clearly would not benefit from ablation with ^131^I, were also excluded.

The 269 patients initially selected were evaluated approximately 16 weeks after thyroidectomy by the measurement of stimulated Tg [after L-T4 withdrawal or administration of rhTSH], TgAb, and neck US. Thirty patients with positive TgAb, and 50 patients with Tg > 5 ng/mL after L-T4 withdrawal or > 2 ng/mL after rhTSH and who were treated with ^131^I were excluded from the study. The data of 136 patients with stimulated Tg ≤ 1 ng/mL, negative TgAb and US without abnormalities have been published previously (7). We report here the results obtained for 53 patients with only slightly elevated stimulated Tg [> 1 ng/mL, but ≤ 5 ng/mL after L-T4 withdrawal or ≤ 2 ng/mL after rhTSH]. These patients also did not receive ^131^I.

The study was approved by the Research Ethics Committee of our institution.

### Follow-up

After stimulated Tg was obtained, which ruled out the need for ablation, the first control assessment was performed approximately 3 months later. The patients were maintained on 0.3 to 2 mIU/L TSH and were followed up by clinical examination, measurement of Tg during L-T4 therapy (Tg/T4) and TgAb at intervals of 6-12 months, and annual neck US.

### Imaging methods

All suspected lesions apparent on the scans (12,13) were evaluated by US-guided fine-needle aspiration biopsy.

#### Assays

Chemiluminescent assays were used for the measurement of Tg [Access (Beckman Coulter, Fullerton, CA; functional sensitivity of 0.1 ng/mL)] and TgAb [Immulite 2000 (Diagnostic Products Corporation, Los Angeles, CA; detection limit of 20 IU/mL; reference value of up to 40 IU/mL) or ARCHITET (Abbott Laboratories, IL, USA; detection limit of 1 IU/mL; reference value of up to 4.11 IU/mL)]. Patients with positive TgAb were excluded.

## RESULTS

### Characteristics of the patients

The characteristics of the patients are shown in Table 1. Twenty-seven patients had tumors > 1 cm and ≤ 4 cm restricted to the thyroid (pT^1^b-2, cN0pNx, M0), whereas 26 patients presented tumors > 4 cm or minimal extrathyroid invasion (pT3, cN0pNx, M0).

**Table 1:** Characteristics of the patients studied

Sex	
Female	42 (79.2%)
Male	11 (20.7%)
Age (years)	14 to 80 (mean: 48.4)
Tumor size	
Range	0.7 to 5 cm
≤ 1 cm	11 (20.7%)
> 1 cm and ≤ 2 cm	20 (37.7%)
> 2 cm and ≤ 4 cm	16 (30.2%)
> 4 cm	6 (11.3%)
Histological subtype	
Classic	39 (73.6%)
Nonencapsulated follicular variant	10 (18.9%)
Hurthle-cell	4 (7.5%)
Minimal extrathyroid invasion (pT3)	20 (37.7%)
Multicentricity of the tumor	17 (32%)
TNM classification	
pT1b, cN0pNx, M0	15 (28%)
pT2, cN0pNx, M0	12 (28%)
pT3, cN0pNx, M0	26 (49%)
Stimulated Tg [range (ng/mL)]	
L-T4 withdrawal (n = 30)	1.4-4.8 ng/mL
rhTSH (n = 23)	1.06-2 ng/mL

Tg: thyroglobulin; rhTSH: recombinant human TSH.

### Follow-up

The time of follow-up ranged from 36 to 96 months (median: 66 months). Among the patients studied, fifty-two continued to present negative US. Lymph node metastases were detected by US in one patient 3 years after thyroidectomy (Table 2) [0/27 patients with tumors ≤ 4 cm restricted to the thyroid and 1/26 (3.8%) patients with tumors > 4 cm or minimal extrathyroid invasion had apparent disease]. Six months after reoperation, this patient had normal neck US and stimulated Tg was < 2 ng/mL in the absence of TgAb. On the basis of these findings, this patient did not receive radioiodine and continues without apparent disease 32 months after reoperation.

**Table 2:** Characteristics of the patient with recurrence

Sex	Female
Age (years)	58
Tumor size (cm)	3.5
Extrathyroid invasion	Yes
Lymph node metastases	No
Multicentricity of the tumor	No
TNM classification	pT3, cN0pNx, M0
Postoperative stimulated Tg	1.6 ng/mL (Tg/rhTSH)
Interval between surgery and recurrence (months)	36
Site of recurrence	Cervical lymph nodes
Positive imaging method	Ultrasonography

Tg: thyroglobulin; rhTSH: recombinant human TSH.

All patients had Tg/T4 ≤ 1 ng/mL in the last assessment. Twenty one patients had undetectable Tg/T4 and 32 patients had detectable Tg ranging from 0.12 to 1 ng/mL (median: 0.3 ng/mL). During follow-up, none of 32 patients with detectable Tg/T4 showed an increase in Tg concentration. When compared to the measurement obtained about 6 months after surgery, Tg concentrations were stable in 12 patients and were reduced in 20.

## DISCUSSION

Low-risk patients with PTC who present stimulated Tg ≤ 1 ng/mL in the absence of TgAb and combined with negative neck US after thyroidectomy do not require ablation with ^131^I (4-9). This Tg level can be achieved in 30-60% of patients submitted to total thyroidectomy (4-11,14,15). Similar to what is observed in patients with stimulated Tg ≤ 1 ng/mL (4,7,9) and reported in previous studies (4,9), the results obtained here suggest that low-risk patients with PTC who present only slightly elevated stimulated Tg combined with negative neck US after thyroidectomy also do not initially require ablation with ^131^I. A weakly positive stimulated Tg after thyroidectomy can represent a small residuum of normal tissue rather than persistent disease. In fact, these patients rarely exhibit ectopic uptake upon RxWBS (5,10,11,14). This group could correspond to nearly 20-30% of low-risk patients (4,5,9,14,15).

To our knowledge, there are only two studies reporting the evolution of patients who were not submitted to ablation because of low postoperative Tg (4,9). In those studies, 61 low-risk patients with Tg between 1 and 5 ng/mL after L-T4 withdrawal did not receive ^131^I and one presented recurrence (4,9). In the present study which included a larger number of patients (n = 53), the recurrence rate was also low (approximately 2%). All patients had Tg/T4 ≤ 1 ng/mL in the last assessment. None of patients with detectable Tg/T4 showed an increase in Tg concentration. Tg concentrations were stable or decreasing, a fact that predicts the absence of long-term disease (16-18). Neck US continued to be negative in all of these patients. Since 50% of recurrences occur in the first 3 years and 80% in the first 5 years (19), the time of follow-up [≥ 36 months in all patients (median: 66 months)] does not seem to significantly compromise the result. The rate of recurrence remained low [1/32 (3.1%)] when only patients with a follow-up time > 5 years were analyzed (unpublished data).

Interestingly, ablation with ^131^I do not guarantee the absence of recurrence in patients with low stimulated Tg after thyroidectomy (14,15). We also observed this fact when reviewing data from 65 patients seen at our institution, who were similar to the present patients (nonaggressive histology; pT1b-3, cN0pNx, M0; Tg between 1 and 5 ng/mL after L-T4 withdrawal) and were submitted to the same follow-up protocol, but who underwent ablation with ^131^I (5). One case of neck recurrence (1.5%) was also detected in this group (unpublished data).

Although undetectable Tg/T4 measured by highly sensitive assays is a predictor of low stimulated Tg, at present, the criterion proposed (4-7,9) and evaluated in this study to spare low-risk patients from ablation would be stimulated Tg. Finally, prophylactic dissection of the cervical lymph nodes was not performed in the present investigation. We believe that, for the selection of patients who can be spared from ablation with ^131^I based on postoperative stimulated Tg, the absence of lymph node metastases can be demonstrated by US and perioperative examination (cN0) and does not require confirmation by prophylactic dissection of the central neck compartment.

## CONCLUSION

The results obtained here suggest that low-risk patients with PTC who present stimulated Tg ≤ 5 ng/mL after L-T4 withdrawal or ≤ 2 ng/mL after rhTSH in the absence of TgAb and combined with negative neck US after thyroidectomy do not initially require ablation with ^131^I. These patients would be followed up by periodic measurement of Tg/T4 and TgAb and neck US, maintaining on 0.5 to 2 mIU/L TSH. Studies involving a larger number of patients are needed to confirm this proposal.
